# Treatment patterns of non-ST-elevation acute coronary syndrome patients presenting at non-PCI centres in the Netherlands and possible logistical consequences of adopting same-day transfer to PCI centres: a registry-based evaluation

**DOI:** 10.1007/s12471-019-1229-2

**Published:** 2019-01-25

**Authors:** N. P. G. Hoedemaker, P. Damman, H. A. Bosker, P. W. Danse, A. H. Liem, B. Geerdes, H. van Laarhoven, R. J. de Winter, The NVVC NSTEMI-ACS project group

**Affiliations:** 1 Heart Center, Department of Cardiology, Amsterdam Cardiovascular Sciences, Amsterdam University Medical Centre, University of Amsterdam, Amsterdam, The Netherlands; 2 Radboud University Medical Center, Nijmegen, The Netherlands; 3 Department of Cardiology, Rijnstate Ziekenhuis, Arnhem, The Netherlands; 4 Department of Cardiology, Franciscus Gasthuis & Vlietland, Rotterdam, The Netherlands; 5 Achmea Health Insurance Company, Zeist, The Netherlands; 6 De Hart&Vaatgroep/Dutch Heart Foundation, The Hague, The Netherlands

**Keywords:** Non-ST-elevation acute coronary syndrome, coronary angiography, PCI, same-day transfer

## Abstract

**Background:**

European Society of Cardiology (ESC) guidelines recommend same-day transfer to a percutaneous coronary intervention (PCI) centre for angiography in high-risk (ESC-HR) patients with non-ST-elevation acute coronary syndrome (NSTE-ACS). We describe the treatment patterns of NSTE-ACS patients presenting at non-PCI centres and evaluate the logistical consequences of adopting same-day transfer.

**Methods:**

From August 2016 until January 2017, all consecutive NSTE-ACS patients presenting at 23 non-PCI centres in the Netherlands were recorded. We built an online case report form in collaboration with the National Cardiovascular Database Registry to collect information on risk stratification by the attending physician, timing and location of angiography, and treatment.

**Results:**

We included 871 patients (mean age 69.1 ± 12.8). 55.8% were considered ESC-HR. Overall, angiography at non-PCI centres was 55.1% and revascularisation was 54.1%. Among ESC-HR patients, angiography at non-PCI centres was 51.4% and revascularisation was 54.9%. Angiography <24 h was 55.6% in patients with angiography at a non-PCI centre and 74.3% in patients with angiography at a PCI-centre. Assuming patients would receive similar treatment, adoption of same-day transfer would increase transfers of ESC-HR patients who undergo PCI (44.3%), but also increases transfers of medically treated patients (36.2%) and patients awaiting coronary bypass artery grafting (9.1%).

**Conclusions:**

In this registry of NSTE-ACS patients at non-PCI centres, the majority of ESC-HR patients underwent angiography at a non-PCI centre. Same-day transfer occurred in one-quarter of the ESC-HR patients, despite guideline recommendation. Nonselective adoption of same-day transfer to a PCI centre would increase transfers of ESC-HR patients who undergo PCI, however, equally increases transfers of patients who are medically treated.

## What’s new

- The 2015 ESC NSTE-ACS guidelines recommend same-day transfer (<24 h) to a PCI centre for angiography in NSTE-ACS patients with elevated troponin levels.
- The majority of Dutch non-PCI centres perform angiography in NSTE-ACS patients, contrary to the guidelines.
- Consequences of adopting same-day transfer in the Netherlands remain unclear.
- In this registry half of the patients presenting at non-PCI centres undergo angiography at a non-PCI centre. Same-day transfer occurred in one-quarter of the patients.
- Non-selective adoption would increase same-day transfers of NSTE-ACS patients who undergo PCI, however, equally increases transfers to PCI centres of patients who are eventually medically treated.

## Introduction

Non-ST-elevation acute coronary syndrome (NSTE-ACS) encompasses a clinical syndrome caused by superimposed thrombus formation due to intracoronary plaque rupture or erosion [1]. Coronary angiography is used to confirm obstructive coronary artery disease and select treatment with percutaneous coronary intervention (PCI), coronary bypass artery grafting (CABG), or medical therapy alone [1]. The European Society of Cardiology (ESC) recommends immediate angiography (<2 h) in patients at very high risk for mortality (ESC-VHR) (e. g. recurrent refractory angina, life-threatening arrhythmia or cardiogenic shock and haemodynamic instability) [1]. Early angiography is recommended in high-risk patients (ESC-HR) presenting with a rise or fall in high-sensitivity cardiac troponin (hs-cTn), or dynamic ST- or T‑wave changes, or a Global Registry of Acute Coronary Events (GRACE) risk score >140 points. In intermediate-risk patients (ESC-IR) the guidelines advise to undergo angiography within 72 h In low-risk patients (ESC-LR), a non-invasive ischaemia detection test is recommended.

Various randomised trials support the use of an early invasive strategy with angiography within 24 h [2, 3]. However, other studies could not demonstrate a benefit of an early invasive strategy versus a more ischemia-driven selective invasive strategy, including patients with elevated cardiac troponin levels [4]. Moreover, recent meta-analyses of contemporary randomised trials could not establish an association between an early invasive strategy and hard clinical outcomes, compared with a delayed strategy [5, 6].

Compared with the 2011 guidelines, the 2015 NSTE-ACS guidelines put more emphasis on same-day transfer to a PCI centre in patients with at least one high-risk criterion (e. g. elevated hs-cTn [2015 NSTE-ACS guidelines, Fig. 6]) [1, 7]. Both the 2011 and 2015 guidelines recommend ‘timely transfer for patients admitted to hospitals without on-site catheterisation facilities’ [1]. However, the guidelines do not specifically comment on ESC-HR patients presenting at non-PCI centres with on-site catheterisation facilities.

In the Netherlands, the majority of non-PCI centres are equipped with chest pain units, cardiac care units, and catheterisation laboratories where NSTE-ACS patients often undergo angiography. Angiographic results are discussed in a Heart Team and, if indicated, patients are referred to an interventional centre for PCI or CABG procedures.

The members if the ACS Working Group of the Netherlands Society of Cardiology (NVVC) remain concerned over the adoption of same-day transfer to PCI centres. They suggested that adoption of same-day transfer may increase transfers of patients with hs-cTn elevation due to type 2 myocardial infarction (MI) or other cardiac and non-cardiac aetiologies [8]. In addition, the possible logistical consequences of adopting same-day transfer for ambulance services and PCI and non-PCI centres remain unclear. The objective of this study was to describe the current treatment patterns (not the outcomes) of NSTE-ACS patients presenting at non-PCI centres in the Netherlands and evaluate the possible logistical consequences of adopting same-day transfer in clinical practice.

## Methods

### Study population and data collection

From August 22th, 2016, until January 31th, 2017, all consecutive patients with a diagnosis or suspicion of NSTE-ACS who presented to 1 of 23 voluntarily participating non-PCI centres in the Netherlands were included (participating hospitals listed below). All hospitals were equipped with catheterisation facilities. Hospitals prospectively recorded in an online form baseline characteristics, medical history, ESC risk category stratified by the attending physicians, Heart Team discussion, and timing of invasive angiography and revascularisation. Cardiologists from the Dutch non-ST-elevation myocardial infarction (NSTEMI)-ACS project group developed and approved this concise form. According to Dutch law no informed consent had to be obtained for this study.

### Data management

The online form was developed and managed by Reports BV (Almere, the Netherlands) and National Cardiovascular Database Registry (NCDR) (Utrecht, the Netherlands). NCDR is an independent Dutch registry with experience in ACS registries [9]. All data were stored anonymously with an encrypted identification number. A trusted third party (Zorg TTP, Houten, the Netherlands) managed the decryption key.

### Definitions

NSTE-ACS was defined as acute angina (or atypical presentation suspected for NSTE-ACS) >20 min at rest in presence of normal ECG findings or ECG patterns associated with NSTE-ACS (ST-segment depression, transient ST-segment elevation, and T‑wave changes). The attending physician used ESC NSTE-ACS risk criteria to stratify patients into risk categories [1]. Time to angiography or revascularisation was defined as time from admission to procedure.

### Statistical analysis

Normally distributed continuous variables were represented by mean and standard deviation. Non-normally distributed variables are shown using median and interquartile range (IQR). Continuous variables we compared with the unpaired *T* test or Mann-Whitney U test, as appropriate and categorical variables with the χ^2^ test. A *p*-value of <0.05 was considered statistically significant. All analyses were performed using SPSS version 24.0 (SPSS Inc, Chicago, IL, USA).

## Results

### Demographics, risk factors, and medical history

**Fig. 1:**
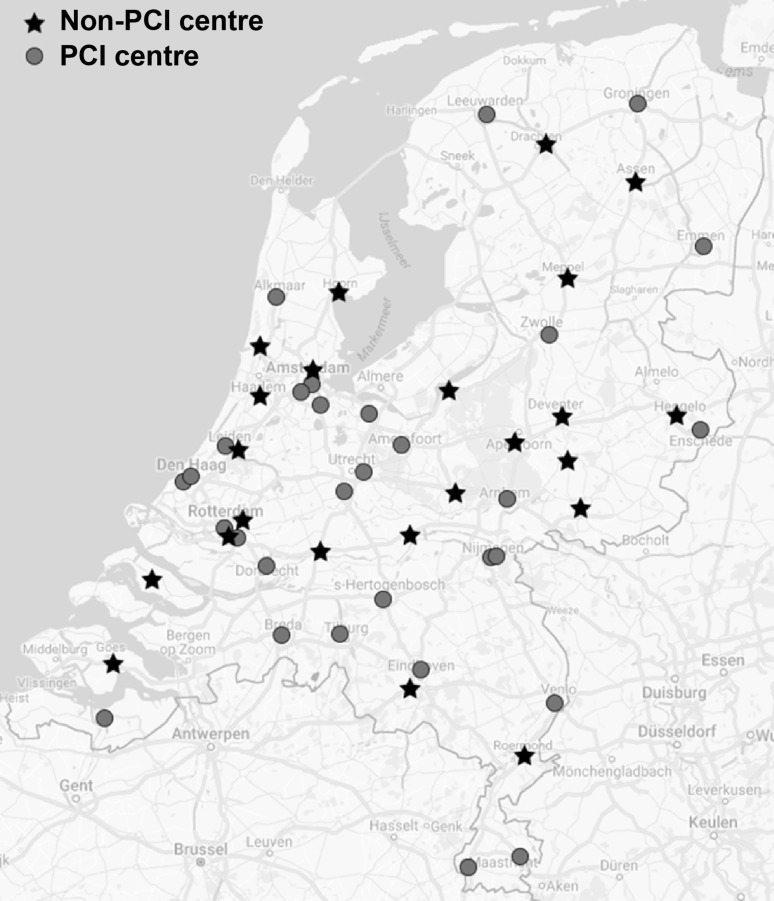
A map of the Netherlands with the participating 23 non-PCI centres and all 30 PCI centres

A total of 871 patients (Tab. 1) from 23 non-PCI centres were included in this registry (Fig. 1). The number of patients per centre ranged between 1 and 86 cases (1 centre recorded 1 patient, all other centres recorded ≥18 patients). The mean age was 69.1 (±12.8) and 36.9% of patients were female. A definite NSTE-ACS diagnosis at admission according to the attending physician was reported in 86.1% of patients. All 23 centres performed on-site coronary angiography.

**Table 1:** Demographics and clinical characteristics

	NSTE-ACS patients
	(*n* = 871)
Age	69.1 ± 12.8
Female	321/871 (36.9)
Definite NSTE-ACS diagnosis	750/871 (86.1)
Admission during weekdays	652/871 (74.9)
Atrial fibrillation	87/871 (10.0)
Diabetes mellitus	156/871 (17.9)
Heart failure	34/871 (3.9)
Renal insufficiency	131/871 (15.0)
Previous AMI	137/871 (15.7)
Previous CABG	75/871 (8.6)
Previous CVA	59/871 (6.8)
Previous PAD	55/871 (6.3)
Previous PCI	170/871 (19.5)

Values are number of cases (%) or mean ± standard deviation.

Renal insufficiency is defined as glomerular filtration rate <60 ml/min/1.73^2.^

*NSTE-ACS* non ST-elevation acute coronary syndrome*, AMI* acute myocardial infarction*, CABG* coronary bypass grafting*, CVA* cerebrovascular accident,* PAD* peripheral artery disease*, PCI* percutaneous coronary intervention

### ESC risk stratification and angiography

**Fig. 2:**
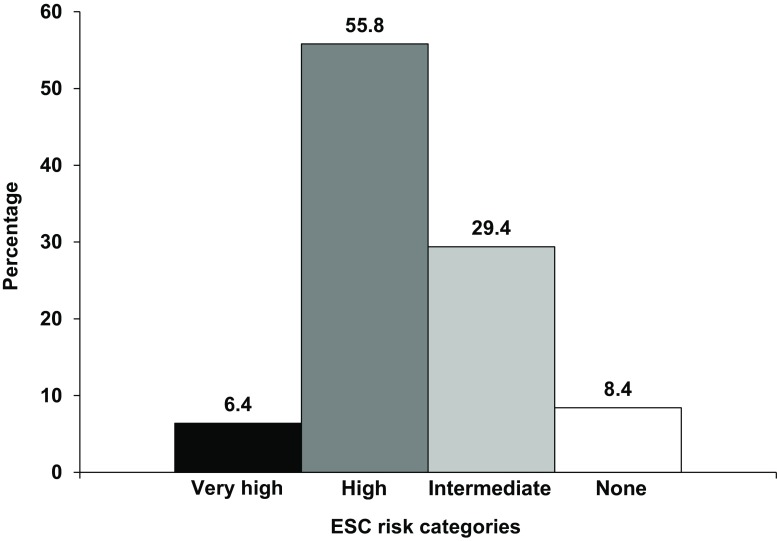
Proportion of ESC risk categories in all 871 NSTE-ACS presentations

The majority of patients (55.8%) were categorised as ESC-HR (Fig. 2). A rise or fall of hs-cTn was the most common criterion in ESC-HR patients (80.0%) (Tab. 2). Overall, 84.7% underwent angiography during hospitalisation (Tab. 3). The median time to angiography was 24 h (IQR: 12–48 h). Overall, the majority of patients (55.1%) underwent angiography at a non-PCI centre, followed by 24.9% of patients who had initial angiography at a PCI centre. Some patients (4.7%) underwent angiography at a non-PCI centre followed by a second angiography at a PCI centre (for additional assessment usually with PCI performed subsequently). The number of patients undergoing angiography per risk category was as follows: 85.7% in ESC-VHR, 81.3% in ESC-HR, 89.5% of ESC-IR patients (Tab. 3). Overall, 90.7% of patients underwent angiography <72 h Angiography was performed within the time frame recommended by the ESC in 57.5% of ESC-VHR (<2 h), 60.8% of ESC-HR (<24 h), in 91.8% of ESC-IR (<72 h) patients.

**Table 2:** ESC risk stratification

	NSTE-ACS patients
	(*n* = 871)
*Very-high-risk criteria*	*(n* *=* *56)*
Haemodynamic instability or cardiogenic shock	1/56 (1.8)
Recurrent or ongoing chest pain refractory to medical treatment	31/56 (55.4)
Life-threatening arrhythmias or cardiac arrest	4/56 (7.1)
Mechanical complications of MI	0/56 (0.0)
Acute heart failure	13/56 (23.2)
Recurrent or dynamic ST-T wave changes	14/56 (25.0)
*High-risk criteria*	*(n* *=* *486)*
Rise or fall in cardiac troponin compatible with MI	389/486 (80.0)
Dynamic ST- or T‑wave changes (symptomatic or silent)	118/486 (24.3)
GRACE score >140	187/486 (38.5)
*Intermediate-risk criteria*	*(n* *=* *256)*
Diabetes mellitus	48/256 (18.8)
Renal insufficiency	42/256 (16.4)
LVEF <40% or congestive heart failure	8/256 (3.1)
Early post-infarction angina	6/256 (2.3)
Prior PCI	36/256 (14.1)
Prior CABG	16/256 (6.3)
GRACE risk score >109 and <140	193/256 (75.4)
*Low-risk score/none of the above*	*(n* *=* *73)*

Values are number of cases (%)

*ESC* European Society of Cardiology*, NSTE-ACS* non ST-elevation acute coronary syndrome*, MI* myocardial infarction*, LVEF* left ventricular ejection fraction*, PCI* percutaneous coronary intervention*, CABG* coronary bypass grafting

**Table 3:** Timing of coronary angiography and revascularisation in ESC risk groups

NSTE-ACS patients (*n* = 871)				
	Overall	Very high	High	Intermediate
	(*n* = 871)	(*n* = 56)	(*n* = 486)	(*n* = 256)
*Coronary angiography*				
Angiography	738/871 (84.7)	48/56 (85.7)	395/486 (81.3)	229/256 (89.5)
Angiography at non-PCI centre	480/871 (55.1)	6/56 (10.7)	250/486 (51.4)	166/256 (64.8)
Angiography at PCI centre	217/871 (24.9)	39/56 (69.9)	123/486 (25.3)	48/256 (18.8)
Angiography at non-PCI and PCI centre^a^	41/871 (4.7)	3/56 (5.4)	22/486 (4.5)	15/256 (5.9)
No angiography	102/871 (11.7)	8/56 (14.3)	72/486 (14.8)	16/256 (6.3)
Unknown	31/871 (3.6)	0/56 (0.0)	19/486 (3.9)	11/256 (4.3)
*Timing of coronary angiography*				
Angiography <2 h	71/636 (11.2)	23/40 (57.5)	34/337 (10.1)	11/196 (5.6)
Angiography 3–24 h	299/636 (47.0)	8/40 (20.0)	171/337 (50.7)	90/196 (45.9)
Angiography <24 h	370/636 (58.2)	31/40 (77.5)	205/337 (60.8)	101/196 (51.5)
Angiography 25–72 h	207/636 (32.5)	5/40 (12.5)	96/337 (28.5)	79/196 (40.3)
Angiography <72 h	557/636 (90.7)	36/40 (90.0)	301/337 (89.3)	180/196 (91.8)
*Heart team*				
Discussed by Heart Team	366/871 (42.0)	12/56 (21.4)	200/486 (41.2)	123/256 (48.0)
*Treatment*				
Revascularisation	471/871 (54.1)	34/56 (60.7)	267/486 (54.9)	142/256 (55.5)
PCI	376/871 (43.2)	30/56 (53.6)	215/486 (44.2)	111/256 (43.4)
CABG	95/871 (10.9)	4//56 (7.1)	52/486 (10.7)	31/256 (12.1)
Medical therapy	311/871 (35.7)	15/56 (26.8)	173/486 (35.6)	87/256 (34.0)
Unknown	89/871 (10.2)	7/56 (12.5)	46/486 (9.5)	27/256 (10.5)
*Treatment after angiography*				
Revascularisation	471/738 (63.8)	34/48 (70.8)	267/486 (67.6)	142/229 (62.0)
PCI	376/738 (50.9)	30/48 (62.5)	215/486 (54.4)	111/229 (48.5)
CABG	95/738 (12.9)	4/48 (8.3)	52/486 (13.2)	31/229 (13.5)
Medical therapy	209/738 (28.3)	10/48 (20.8)	104/486 (26.3)	64/229 (27.9)
Unknown	58/738 (7.9)	4/48 (8.3)	24/486 (6.1)	23/229 (10.0)
*Time indicators (hours)*				
Time to coronary angiography	24.0 (12.0–48.0) 636 pts	2.0 (1.0–24.0) 40 pts	24.0 (10–48.0) 337 pts	24.0 (16.0–48.0) 196 pts
Time to PCI	65.0 (21.8–120.0) 318 pts	2.0 (1.0–33.0) 24 pts	72 (24.0–120.0) 182 pts	66.0 (24.0–120.0) 92 pts
Time to CABG	216.5 (163.8–322.5) 24 pts	217.0 (229.5–) 2 pts	216 (169.5–381.0) 13 pts	175.0 (78.5–298.8) 8 pts

Values are number of cases (%) or median with interquartile range; time in hours

All patients were first admitted to a non-PCI centre. Coronary angiography at PCI centre indicates transfer from a non-PCI centre to a PCI centre with initial angiography at the PCI centre

*ESC* European Society of Cardiology*, NSTE-ACS* non ST-elevation acute coronary syndrome*, PCI* percutaneous coronary intervention*, CABG* coronary bypass grafting*, pts* patients

^a^initial angiography at a non-PCI centre with additional angiography at a PCI centre for additional assessment (e. g. additional angiographic assessment, sometimes followed by intracoronary fractional flow reserves and/or coronary flow reserve measurements)

### Treatment

In total, 54.1% of patients underwent revascularisation (43.2% PCI and 10.9% CABG) and 35.7% were treated with medical therapy (Tab. 3). Treatment was not recorded for 10.2% of patients. Revascularisation was performed in 54.9% of ESC-HR patients. Overall, 28.3% of patients who underwent angiography were treated with medical therapy.

### Angiography at a non-PCI centre (non-transferred) versus angiography at a PCI centre (transferred)

**Fig. 3:**
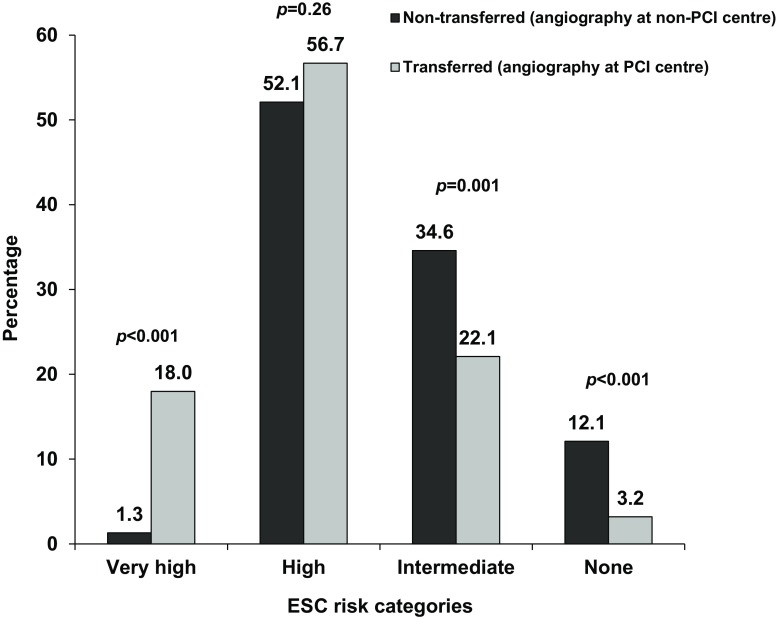
Differences in percentage of each ESC risk category displayed for patients undergoing angiography at non-PCI centres (*non-transferred*) and patients undergoing angiography at PCI centres (*transferred*)

Baseline characteristics of patients who underwent angiography at a non-PCI centre (non-transferred) or at a PCI centre (transferred) were mostly similar, however, non-transferred patients were older (67.4 ± 11.3 vs. 65.1 ± 12.7, *p* = 0.02). The distribution of risk categories was markedly differed among non-transferred and transferred patients, except for ESC-HR patients (Fig. 3). Median time to angiography was shorter in transferred patients (10 h, IQR: 2–24) compared with patients who underwent angiography at a non-PCI centre (24 h, IQR: 16.8–48). Transferred patients were less often discussed by the Heart Team (57.9% vs. 26.7%, *p* < 0.001). Revascularisation was 58.3% in non-transferred patients and 71.0% in transferred patients (*p* < 0.001).

### High-risk patients

All 23 non-PCI centres admitted ESC-HR patients, of which 22 centres (95.7%) performed on-site angiography in these patients. Non-transferred ESC-HR patients were older (68.7 (±11.7) vs. 64.7 (±13.1), *p* = 0.003), without other baseline differences. Angiography <24 h was more common in transferred ESC-HR patients (74.3% vs. 55.6%, *p* = 0.004). Notably, angiography <2 h was higher among transferred ESC-HR patients (23.0% vs. 6.2%, *p* < 0.001) and angiography <3–24 h was similar (transferred: 51.3% vs. non-transferred: 49.4%, *p* = 0.77). Heart Team discussion was more common in non-transferred ESC-HR patients (58.4% vs. 30.9%). Revascularisation was higher in transferred ESC-HR patients (74.8% vs. 62.0%, *p* = 0.002).

### Possible consequences of adopting same-day transfer to a PCI centre

**Fig. 4:**
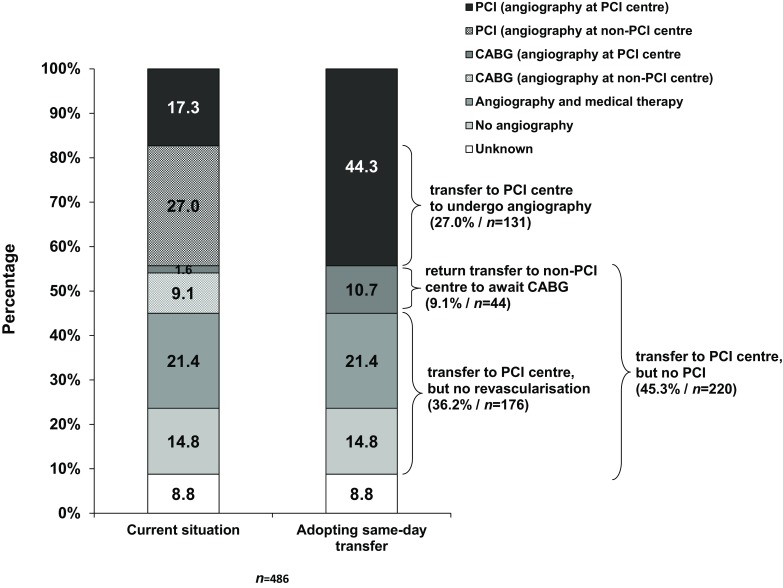
The current situation (*left bar*) and possible logistical consequences of adopting same-day transfer to undergo angiography at a PCI centre, as recommended by the 2015 NSTE-ACS ESC guidelines (*right bar*) (*PCI* percutaneous coronary intervention, *CABG* coronary artery bypass grafting, *NSTE-ACS* non-ST-elevation acute coronary syndrome, *ESC* European Society of Cardiology)

Angiography and revascularisation status was known in 91.2% of ESC-HR patients (Fig. 4). In our current observation, 14.8% of ESC-HR did not undergo angiography and 21.4% were medically treated after angiography. Of the patients who underwent CABG, the majority (9.1%) underwent angiography at a non-PCI centre and 1.6% were directly transferred to a PCI centre for angiography. One-quarter of patients (27.0%) underwent angiography at a non-PCI centre and were later referred for PCI and 17.3% of patients were directly transferred to a PCI centre for angiography with subsequent PCI.

Assuming patients would receive similar treatment as observed in this registry, adoption of same-day transfer to a PCI centre would increase transfers to PCI centres of ESC-HR patients who undergo PCI (44.3%). However, 36.2% of the ESC-HR patients would be transferred to a PCI centre, but would be medically treated and not undergo PCI. Patients who would undergo CABG, but would otherwise be awaiting surgery at a non-PCI centre (9.1%), may need to be transferred back from the PCI centre to their referring non-PCI centre to await surgery.

## Discussion

In this registry of NSTE-ACS patients presenting at 23 non-PCI centres in the Netherlands, we found that the majority of patients (55.8%) were classified as ESC-HR, mostly based on elevated hs-cTn levels. Our findings highlight that angiography of ESC-HR NSTE-ACS patients at non-PCI centres is still common (51.4%) and same-day transfer to a PCI centre only occurs in one-quarter of the ESC-HR patients, despite guideline recommendations. The revascularisation rate in ESC-HR patients was 54.9%. Assuming patients would receive similar treatment as observed in this registry, adopting same-day transfer of ESC-HR NSTE-ACS patients presenting at non-PCI centres would lead to an increase of transfers of patient who undergo PCI. However, this would also result in at least an equal number of transfers of NSTE-ACS patients treated with medical therapy alone.

The optimal timing of angiography and revascularisation in NSTE-ACS has been extensively studied. Since NSTE-ACS is a heterogeneous condition, the expected benefit from early angiography depends greatly on the severity of the symptoms and the patient’s risk profile. Therefore, timing of angiography is based on risk stratification [1]. The recommendation of same-day transfer aims to minimize the delay of angiography (and revascularisation) in ESC-HR NSTE-ACS patients. The 2015 guidelines put more emphasis on same-day transfer of ESC-HR patients to a PCI-centre, compared with earlier guidelines [1]. However, both the 2011 and 2015 ESC NSTE-ACS guidelines are largely based on the same studies [1, 3, 7, 10–13]. In addition, two more studies were added to support the 2015 guidelines [14, 15]. Although various studies cited by the guidelines could not prove a benefit on hard clinical endpoints with an early invasive strategy (<24 h), early angiography did reduce recurrent ischaemia and shorten length of hospitalisation [13].

Our findings show that among non-transferred and transferred ESC-HR patients, patients who were directly transferred to a PCI centre had higher rates of immediate angiography (<2 h) and revascularisation. Additionally, they were less often discussed by a Heart Team compared with non-transferred ESC-HR patients. This may indicate that the attending physician at the non-PCI centre used clinical assessment, beyond ESC risk stratification, to refer some ESC-HR patients for immediate angiography (possibly followed by PCI) at a PCI centre and considered other ESC-HR patients for angiography at a non-PCI centre. Please note, non-transferred patients were significantly older than transferred patients.

Based on the treatment patterns in our registry and assuming similar treatment, 44.3% of ESC-HR patients underwent PCI. If non-selective same-day transfer of ESC-HR patients were to be adopted, there would be an additional 27% of transfers to a PCI centre of patients who undergo PCI. On the other hand, 36.2% of ESC-HR patients were medically treated and may have a similar benefit of receiving optimal medical therapy either at a non-PCI centre or a PCI centre. It raises the question whether the potential pros of same-day transfer outweigh the cons.

We believe the ideal timing (and location) of angiography in ESC-HR NSTE-ACS patients, needs to be determined in a randomised trial that involves hs-cTn assays, radial artery access, drug-eluting stents, and novel P2Y12 inhibitors. For now, identifying the NSTE-ACS patients who might benefit from early angiography and same-day transfer remains important. A registry of 1,500 ESC-HR NSTE-ACS patients with elevated hs-cTn levels without additional risk factors admitted to German chest pain units, showed similar rates of death, MI, or stroke at 4 months if patients were treated with either medical therapy, PCI <24 h, or PCI >24 h [16]. In the same study ESC-HR NSTE-ACS patients who were at a higher risk (e. g. GRACE score >140), PCI was associated with improved outcomes compared with conservative treatment. Although these results are merely hypothesis-generating, it suggests research should focus on selecting the right patients for same-day transfer, rather than non-selectively adopting same-day transfer of patients with elevated hs-cTn levels.

Currently, the attending physician at a non-PCI centre triages patients who present with chest pain suspected for NSTE-ACS. Several studies were conducted to evaluate pre-hospital triage of patients with suspected NSTE-ACS [17, 18]. Point-of-care troponin T testing by paramedics appears to be feasible [19]. In the NONSTEMI trial, pre-hospital troponin testing was used to diagnose NSTE-ACS, whether immediate or delayed angiography after point-of-care troponin testing benefits outcomes remains to be determined [18]. The FamouS Triage study used the modified HEART score including point-of-care hs-cTn testing in the pre-hospital setting. Patients with a modified HEART score of 0–3 did not develop any events during follow-up and could be safely ruled-out for MI by ambulance personnel [20]. The role of computerised tomography (CT) coronary angiography in NSTE-ACS patients will be further determined in the RAPID-CTCA study, which compares early CT coronary angiography with standard care with invasive angiography in 2,500 patients with suspected NSTE-ACS and elevated hs-cTn levels [21]. The first results are expected in 2019.

### Strengths & limitations

This study presents observational data from a selection of non-PCI centres and offers a perspective of NSTE-ACS care in the Netherlands. There are some limitations. First, 23 non-PCI centres voluntarily recorded data, therefore, these finding should be generalised with caution. Second, the design of this study was concise and with a modest dataset. We did not record the process of clinical decision-making for angiography at a PCI or a non-PCI centre. Third, we could not include the whole study population for all analyses due to missing data. Fourth, we could not include discharge medication, mortality, or clinical follow-up and cannot comment on clinical outcomes of same-day-transfer based on these data.

## Conclusion

In this registry of NSTE-ACS patients at non-PCI centres, most ESC-HR patients underwent angiography at a non-PCI centre and same-day transfer occurred in one-quarter of the ESC-HR patients, despite guideline recommendations. Non-selective adoption of same-day transfer to a PCI centre would increase the number of transfers of ESC-HR patients who undergo PCI, however, equally increases the number of transfers of ESC-HR patients who are medically treated. A randomised trial in a contemporary setting is warranted to determine the benefit of early angiography (and same-day transfer) in NSTE-ACS patients with elevated hs-cTn levels.

## Appendix

### NVVC NSTEMI-ACS project group

R.J. de Winter (chair), H.A. Bosker, P.W. Danse, A.H. Liem, B. Geerdes (external member), H. van Laarhoven (external member).

### List of participating centres

Admiraal de Ruyter Ziekenhuis, Goes (P.S. Monraats) – Alrijne Ziekenhuis, Leiderdorp (P.E.J. Pol) – Beatrixziekenhuis, Gorinchem (J.G.J. Milhous) – BovenIJ Ziekenhuis, Amsterdam (F.M. Sandt) – Deventer Ziekenhuis, Deventer (A. van der Sluis) – Gelre Ziekenhuizen, Apeldoorn (D.M. Nicastia) – Gelre Ziekenhuizen, Zutphen (P. Schaardenburgh) – IJsselland Ziekenhuis, Capelle aan den IJssel (J.J.H. Bennik) – Ikazia Ziekenhuis, Rotterdam (M.E. Emans) – Isala Diaconessenhuis, Meppel (J.H. Geertman) – Laurentius Ziekenhuis, Roermond (C.J.P.J. Werter) – Maxima Medisch Centrum, Veldhoven (F. Rosestraten) – Nij Smellinghe, Drachten (J.J. Graaf) – Rode Kruis Ziekenhuis – Beverwijk (I. Westendorp) – Slingeland Ziekenhuis, Doetinchem (D.A.A.M. Schellings) – Spaarne Gasthuis, Hoofddorp (A.J. Voogel) – Van Weel-Bethesda Ziekenhuis, (T. Baks) – Westfriesgasthuis Hoorn (C.L. Janus), Wilhelmina Ziekenhuis, Assen (I.J. van Eede) – Ziekenhuis Gelderse Vallei, Ede (R.J. Walhout) – Ziekenhuis Rivierenland, (M.J. Asselman) – Ziekenhuis St. Jansdal, Harderwijk (L.J. Klein) – Ziekenhuisgroep Twente, Hengelo (H.J. Kruik).
